# Active wearable device utilization improved physical performance and IGF-1 among community-dwelling middle-aged and older adults: a 12-month prospective cohort study

**DOI:** 10.18632/aging.203383

**Published:** 2021-08-03

**Authors:** Wei-Ju Lee, Li-Ning Peng, Ming-Hsien Lin, Ching-Hui Loh, Liang-Kung Chen

**Affiliations:** 1 Aging and Health Research Center, National Yang Ming Chiao Tung University, Taipei, Taiwan; 2 Department of Family Medicine, Taipei Veterans General Hospital Yuanshan Branch, Yilan County, Taiwan; 3 Center for Geriatrics and Gerontology, Taipei Veterans General Hospital, Taipei, Taiwan; 4 Center of Health and Aging, Hualien Tzu Chi Hospital Buddhist Tzu Chi Medical Foundation, Hualien County, Taiwan; 5 Superintendent Office, Taipei Municipal Gan-Dau Hospital, Taipei, Taiwan

**Keywords:** walking speed, wearable device, average steps, community-dwelling older adults

## Abstract

Wearable devices provide real-time and patient-powered data that enable the development of personalized health promotion and management programs. This study aimed to explore the clinical benefits of using the wearable device and to examine associated factors, utilization patterns on health status. 319 community-living adults aged 50-85 years were enrolled and clinically followed for 12 months. Participants were categorized into 3 groups based on the wearable device utilization patterns (active: >30 days of use, non-active: <3 days of use, usual: 3-30 days of use). 128 (40.1%) and 98(30.7%) were active and usual wearable device users, and no significant differences in the baseline demographic characteristics and functional status were noted across groups. Higher cognitive performance was significantly associated with the wearable device use (OR: 1.3,95%CI: 1.1-1.5, p=0.005). Multivariable linear regression showed that 0.16 m/s increase in walking speed among active users, which was significantly higher than non-active users (p=0.034). Compared to usual users, active users had higher average daily, weekday, and holiday step counts. The walking speed increased for 0.03 m/s when participants walked 1,000 more daily step counts (p=0.020). Active use of wearable devices substantially increased walking speed, which suggested better functional outcomes and survival benefits in the future.

## INTRODUCTION

Advanced development of internet telecommunication technologies (ICT) enables clinicians and healthcare professionals to collect real-time information through wearable biosensors that further changes healthcare services and healthy lifestyles. The integration of electronic health records and wearable devices may overwhelmingly modify the disease diagnosis, treatment and care management of clinical conditions. The World Health Organization’s Global Observatory recognized the roles of mobile devices in supporting medical and public health practice to collect health data, to support diagnosis, to monitor progress, and to promote health promotion [1]. The advantage of real-time and person-powered data nature of wearable devices promotes integration of daily lifestyle conditions in disease diagnosis, health promotion, and personalized care planning that echoes the concepts of precision medicine [2, 3].

Although a great variety of parameters have been developed to measure health, the usual walking speed is a well-established and widely-recognized biomarker to predict adverse health outcomes for older adults [4]. Walking is a complex physical performance interconnected with musculoskeletal, neural systems, and other organ systems. Usual walking speed is a simple functional measure to predict mortality [5], cognitive impairment [6], and disability [7]. Given its nature as a quick, simple and reliable assessment tool for functional ability of older adults, walking speed has been recommended as the indicator for physical health and successful aging [7]. A study of 1925 patients with osteoarthritis showed that intensive walking training significantly prevented walking speed slowing [8], which suggested the potentials to improve or maintain walking speed through appropriate training even on those with orthopedic conditions. Wearable devices have garnered extensive research attentions as a safe, reliable and cost-effective approach to track physical activities [9, 10]. Moreover, the use of wearable devices may modify users’ behavior and enhance their physical activities through self-monitoring and reinforcement [11]. Using wearable devices in health care systems may shift the traditional provider/organization-centric service delivery model to person-centered approach that empowers individuals to carry on healthy lifestyles. Although the potential benefits of using wearable devices for healthy lifestyles have been reported, the effects of wearable devices on health outcomes were inconsistent [12–16]. It has been reported that using wearable devices substantially improved daily sensatory behavior of workers [13], and also increased daily walking steps of older adults [14]. However, using wearable devices did not show improvement in walking speed in the subacute rehabilitation setting [15]. A study of 350 older women aged≥75 years reported that moderate to vigorous physical activities prevented walking speed slowing [16], but those activities were not triggered by the utilization of wearable devices. Moreover, a systematic review of six published studies did not identify significantly favorable effects of using wearable devices on chronic conditions management and related health outcomes [12]. Hence, this study aimed to evaluate the potential health benefits of wearable device use among community-dwelling middle-aged and older persons during the 12-month follow-up. We hypothesized that reciprocal self-monitoring and reinforcement may enable behavior changes, prevent functional declines, and improve health outcomes through a longer follow-up period.

## RESULTS

**Figure 1:**
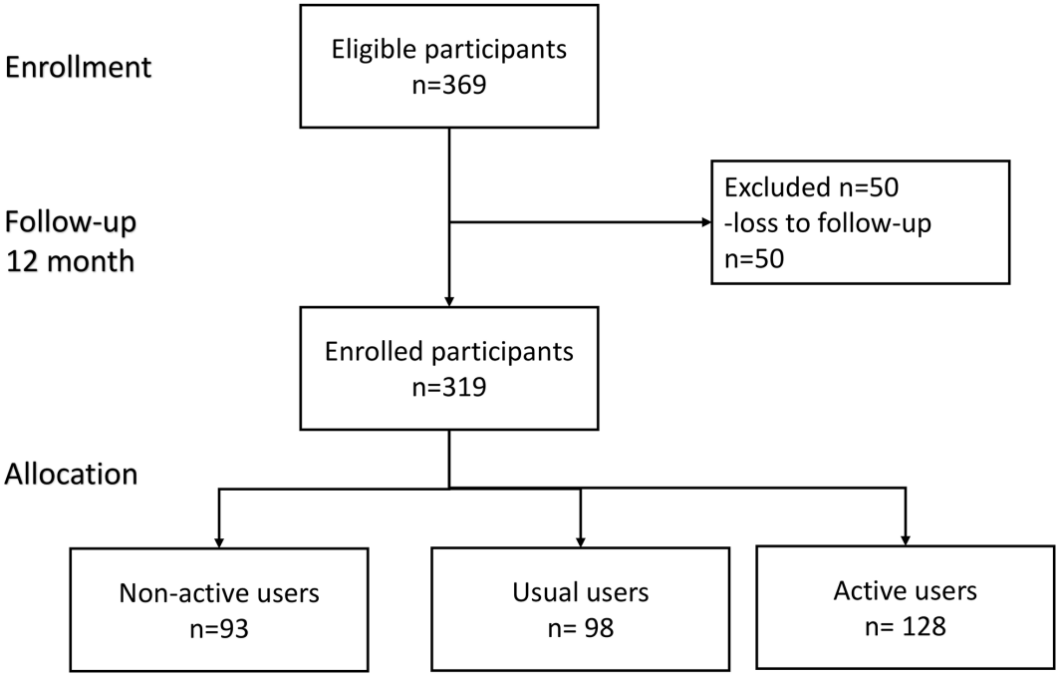
Flowchart diagram of participants in this study.

**Figure 2:**
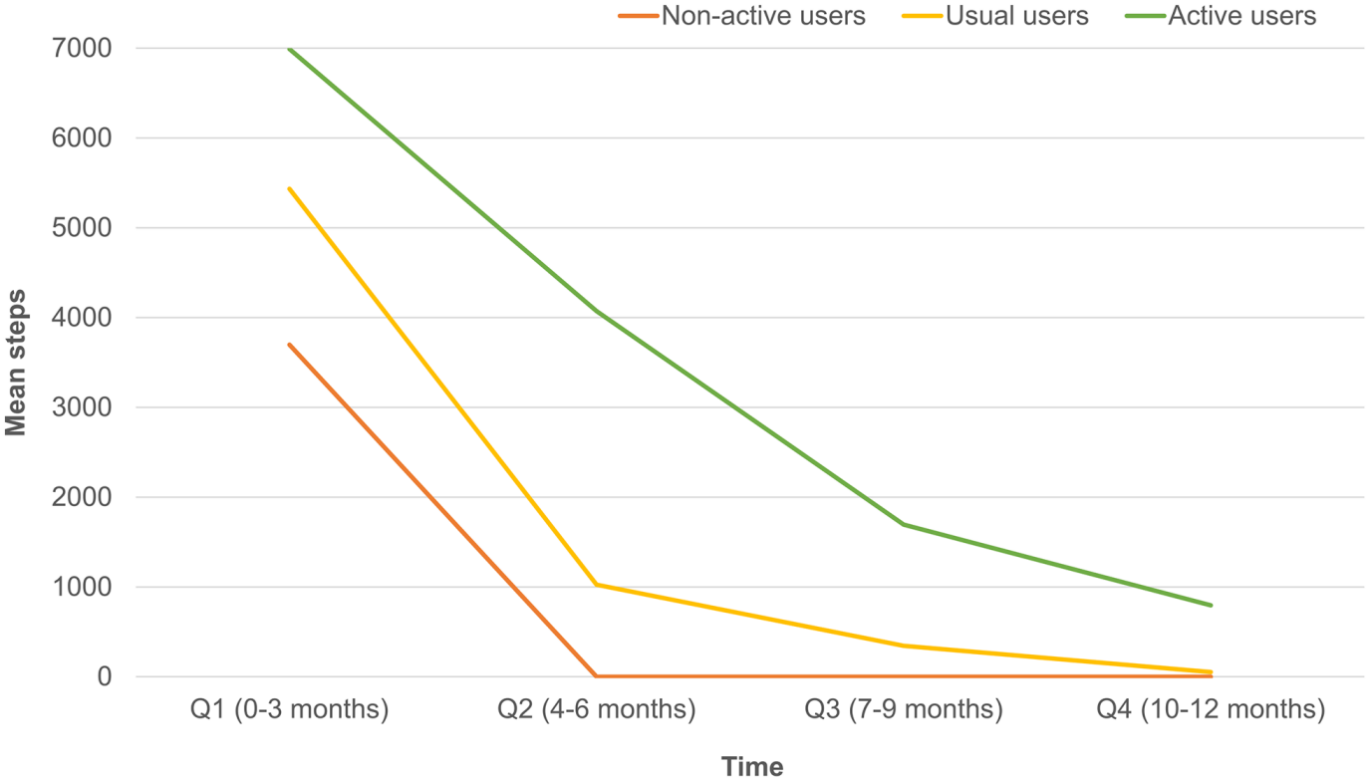
Differences of mean daily steps by quarters in non-active, usual and active users.

Overall, 369 participants were enrolled for study, and 319 (mean age: 64.9±6.6 year, 32.3 % male) of them completed a 12-months follow-up (Figure 1); 128 (40.1%) out of the 319 participants were classified as active users. Table 1 summarized comparisons of demographic characteristics, functional assessments, cardiovascular parameters and age-related hormones between groups. Compared to non-active users, active users had better nutrition(p=0.046) and cognitive performance(p=0.006), higher T-score of bone mineral density (*p* for trend =0.020), lower serum levels of ACTH (*p* for trend =0.005), higher serum levels of IGF-1 (*p* for trend < 0.001), lower serum levels of AST (*p* for trend =0.007), and lower urine microalbumin-to-creatinine ratio (*p* for trend =0.036). However, the appendicular muscle mass was similar to non-active users. Figure 2 showed mean declines of daily steps among participants of the three groups during the study period. Table 2 summarized changes of physical and functional measurements during the study period based on the utilization patterns of the wearable device. Multivariable linear regression showed the changes of walking speed over 12 months of active users was 0.16 m/s higher than that of non-active users, but similar in handgrip strength, 5-time sit-to-stand test and other functional measurements like MNA-SF, CES-D, MoCA, SMAF, and blood pressure measurements. ROC analysis showed the average daily steps of 7,008 was the most optimal cutoff to prevent walking speed slowing in the 12-month period (sensitivity 0.44, specificity 0.68, c-statistics 0.53).

**Table 1:** Characteristics of participants by wearable device.

**Characteristics: data show mean ± SD or number (%)**	**Non-active user (n=93)**	**Usual user (n=98)**	**Active user (n=128)**	**p value**
Age(years)	66.0±7.2	64.6±6.6	64.3±6.1	0.135
Men	33(35.5)	30(30.6)	40(31.2)	0.730
Education (years)	14.2±3.5	13.6±3.2	14.5±3.1	0.181
**Body composition**				
Body height (cm)	161.0±7.9	159.5±8.8	158.6±7.4	0.098
Body weight (kg)	61.1±10.8	60.2±10.6	60.7±10.4	0.859
Body mass index (kg/m^2^)	23.5±3.4	23.6±3.1	24.0±3.2	0.449
Appendicular skeletal muscle (kg)	17.1±4.1	16.8±4.0	17.1±4.2	0.922
Appendicular skeletal muscle/height^2^ (kg/m^2^)	6.5±1.1	6.5±1.0	6.7±1.2	0.192
Bone marrow density (g/cm2)	2.4±0.6	2.5±0.5	2.5±0.4	0.553
T-score	-1.7±1.1	-1.5±1.2	-1.4±1.1	0.018*
**Functional assessment**				
Charlson Comorbidity index	0.7±1.4	0.6±1.1	0.7±1.1	0.926
SMAF	-0.1±0.3	-0.1±0.3	0.0±0.2	0.456
MNA-SF	12.9±1.3	13.1±1.1	13.2±1.0	0.042*
CESD	3.1±5.5	2.5±3.4	2.6±4.4	0.421
MoCA	26.9±2.3	27.5±2.1	27.8±2.0	0.006*
**Physical assessment**				
Walking speed (m/s)	1.9±0.6	1.8±0.6	1.8±0.4	0.061
Grip strength (kg)	28.7±8.7	28.0±8.0	27.9±8.2	0.500
Five Chair Time (second)	8.8±2.2	8.8±2.4	9.0±1.9	0.500
**Cardiovascular parameters**				
Systolic pressure (mmHg)	128.9±16.5	126.3±18.8	131.7±20.2	0.274
Diastolic pressure(mmHg)	78.2±9.1	77.9±9.7	79.7±10.6	0.260
Heart rate (bpm)	68.2±10.5	68.3±8.5	69.8±10.0	0.204
Uric acid(mg/dL)	5.4±1.2	5.5±1.4	5.6±1.2	0.745
Fasting glucose (mg/dL)	95.6±15.0	95.1±12.2	97.6±15.2	0.356
HbA1C (%)	5.7±0.4	5.7±0.4	5.8±0.6	0.570
Insulin (mIU/L)	8.9±6.7	8.9±5.1	8.8±4.0	0.984
HOMA-IR	2.2±1.7	2.2±1.4	2.2±1.1	0.996
Cholesterol (mg/dL)	202.1±31.9	205.5±33.6	203.0±31.5	0.746
Triglyceride (mg/dL)	108.3±84.2	106.3±49.9	112.8±56.7	0.738
High density lipoprotein cholesterol (mg/dL)	62.7±17.1	60.2±14.0	58.9±15.1	0.199
Low density lipoprotein cholesterol (mg/dL)	115.9±28.2	120.9±29.0	119.7±28.0	0.447
high sensitive CRP (mg/L)	1.5±2.7	1.8±2.6	1.7±2.4	0.604
homocysteine (mcmol/L)	13.5±4.8	13.0±4.0	13.0±3.5	0.600
Neutrophil to lymphocyte ratio	1.8±0.8	1.8±0.8	1.9±0.9	0.600
Platelet to lymphocyte ratio	7.5±2.8	7.0±2.6	7.6±3.2	0.798
**Age related hormone**				
ACTH (pmol/L)	19.6±11.7	16.9±8.0	16.0±7.4	0.013*
Cortisol (mcg/dL)	10.9±4.2	10.0±4.1	10.5±3.7	0.258
Growth hormone (ng/mL)	1.7±1.7	1.3±1.5	1.7±1.9	0.295
Insulin-Like Growth Factor 1 (ng/ml)	118.4±42.2	130.7±52.8	142.7±51.8	0.002**
DHEAS(μg/dL)	114.2±63.9	118.9±76.6	126.1±74.1	0.462
Testosterone (ng/dL)	183.8±220.0	159.3±209.4	149.3±183.5	0.450
Sex hormone binding protein (nmol/L)	74.9±42.4	67.3±36.4	63.8±35.5	0.097
**General biochemistry data**				
WBC (x10^3/uL)	5.4±1.1	5.2±1.4	5.5±1.3	0.764
Hemoglobin (g/dL)	14.0±1.3	13.9±1.4	13.9±1.2	0.838
Platelet (x10^3/uL)	240.0±49.7	231.9±50.3	240.2±56.2	0.975
Neutrophil (%)	56.1±9.3	56.4±8.3	57.6±8.6	0.208
Lymphocyte (%)	34.4±8.7	35.4±8.3	34.1±8.2	0.816
AST (U/L)	26.6±7.5	25.9±6.2	24.3±5.0	0.008**
ALT(U/L)	24.8±12.8	24.9±10.1	23.0±7.4	0.186
Albumin (g/dL)	4.5±0.2	4.5±0.2	4.5±0.2	0.769
Globulin(g/dL)	2.9±0.3	2.9±0.3	2.9±0.4	0.805
Blood Urea Nitrogen (mg/dL)	16.4±4.6	15.6±3.2	15.5±2.8	0.117
Creatinine(mg/dL)	0.8±0.2	0.8±0.2	0.8±0.2	0.337
eGFR (mL/min/1.73 m^2^)	89.4±19.8	91.1±16.2	89.7±16.3	0.773
Urine microalbumin creatinine ratio	51.5±177.5	16.4±29.6	19.8±57.9	0.035*

*denotes p<0.05; **denotes p<0.01, bold type denotes statistical significance.

SMAF denotes the Functional Autonomy Measurement System; MNA-SF denotes Short-Form Mini-Nutritional Assessment; CESD denotes Center for Epidemiologic Studies Depression Scale; MoCA denotes the Montreal Cognitive Assessment ACTH denotes; Adrenocorticotropic hormone; DHEA denotes Dehydroepiandrosterone-sulfate; AST denotes aspartate Aminotransferase; ALT denotes alanine aminotransferase.

**Table 2:** Multiple linear regression to explore associations between wearable device users and 12-month walking speed changes.

**12-month differences**	**Usual user (vs. non-active user)**			**Active user (vs. non-active user)**		
	**Coefficients**	**Standard error**	***p***	**Coefficients**	**Standard error**	***p***
**Physical assessment**						
Walking speed (m/s)	0.10	0.08	0.228	0.16	0.08	0.034*
Hand grip strength (kg)	0.16	0.48	0.743	0.10	0.45	0.828
Five chair stand time (second)	-0.30	0.33	0.366	-0.32	0.31	0.305
Number of frail phenotypes	0.17	0.11	0.131	0.10	0.10	0.354
**Functional assessment**						
SMAF	0.03	0.05	0.532	-0.05	0.05	0.226
MNA-SF	-0.06	0.19	0.751	0.01	0.18	0.935
CESD	-0.13	0.48	0.796	-0.20	0.46	0.655
MoCA	-0.36	0.34	0.289	-0.61	0.32	0.054
**Blood pressure measurements**						
Systolic pressure (mmHg)	0.88	2.07	0.670	-0.77	1.95	0.693
Diastolic pressure(mmHg)	0.15	1.26	0.905	-1.13	1.19	0.343
Heart rate (bpm)	1.66	1.58	0.293	1.00	1.49	0.500

*denotes p<0.05; bold type denotes statistical significance.

SMAF denotes the Functional Autonomy Measurement System; MNA-SF denotes Short-Form Mini-Nutritional Assessment; CESD denotes Center for Epidemiologic Studies Depression Scale; MoCA denotes the Montreal Cognitive Assessment.

Compared to usual users, active users had significantly higher average daily walking steps, higher weekday and holiday daily walking steps, lower weekday and holiday low step ratios, and higher weekday high step ratio (Table 3). Table 4 showed results of multiple linear regressions on associations between individual parameters obtained from the wearable devices and changes of walking speed. Among all parameters, high holiday step ratio had higher incremental walking speed for 0.5 m/s. Stepwise multinomial logistic regression showed that higher cognitive performance (Odds ratio (OR): 1.3, 95%CI: 1.1-1.5, p=0.005), and fewer education years (OR:0.89, 95%CI: 0.90-0.98, p=0.022) were significantly associated with active wearable device use (Supplementary Table 1). A sensitivity analysis done by using multinomial logistic regression adjusted for age, sex, education, CCI affirmed that MoCA performance (OR: 1.18, 95%CI: 1.02-1.36, p=0.028) was significantly associated with active wearable device use.

**Table 3:** Parameters of wearable devices between usual and active users.

	**Usual + active users**	**Usual users**	**Active users**	***p* value**
n	226	98	128	
Average steps (per 1000 steps)	6.3±3.2	5.5±3.1	6.9±3.1	0.001**
Average weekday steps (per 1000 steps)	6.5±3.3	5.6±3.2	7.1±3.2	0.001**
Average holiday steps (per 1000 steps)	5.8±3.4	5.2±3.7	6.3±3.0	0.010*
Weekday low step ratio	0.3±0.3	0.4±0.3	0.2±0.2	<.001**
Holiday low step ratio	0.3±0.3	0.4±0.4	0.3±0.2	0.007**
Weekday high step ratio	0.2±0.2	0.2±0.2	0.3±0.2	0.048*
Holiday high step ratio	0.2±0.2	0.2±0.3	0.2±0.2	0.510

*denotes p<0.05; **denotes p<0.01, bold type denotes statistical significance. Data showed mean ± SD or number (%).

**Table 4:** Associations between parameters of wearable device and 12-month walking speed changes.

	**Coefficients**	**Standard error**	***p***
Average steps (per 1000 steps)	0.03	0.01	0.020*
Average weekday steps (per 1000 steps)	0.02	0.01	0.045*
Average holiday steps (per 1000 steps)	0.03	0.01	0.007**
Weekday low step ratio	-0.15	0.14	0.290
Holiday low step ratio	-0.25	0.12	0.040*
Weekday high step ratio	0.30	0.14	0.030*
Holiday high step ratio	0.50	0.14	<.001**

Multiple linear regression adjusted for age, sex, education, body mass index explored individual parameter and 12-month changes of walking speed.

*denotes p<0.05; **denotes p<0.01, bold type denotes statistical significance. Charlson comorbidity index, CES-D, and SMAF. Bold type denotes statistical significance.

## DISCUSSION

The results of the current study clearly identified potential benefits of using wearable devices without specific physical activities programs in the middle-aged and older adults that the utilization of wearable devices significantly prevented walking speed slowing in a 12-month period. Among all participants, weekend warriors (higher high-holiday step ratio) were more likely to have faster walking speed. In general, mean walking steps in weekdays were higher than that in holidays, but active users had higher mean step counts in both weekdays and holidays. Compared to non-active users, cognitive performance was better in active users. Results of this study implied that people with better health and healthy lifestyles were also more likely to use the wearable devices. However, it is also possible that active use of wearable devices may improve users’ lifestyles and their functional performance.

Until now, only a few studies have evaluated the roles of wearable devices utilization in association with health outcomes of older adults. A study of 54 older adults ≥70 years from the primary care setting using pedometers for 12 weeks showed high acceptability and compliance for wearable devices but no significant differences in their step counts [17]. Another study of 130 overweight or obese participants with multimorbidity showed significant improvement of walking speed of 0.08 m/s in 8 weeks goal-setting interventions period [18]. Two other studies echoed the findings, which showed pedometer use with goal-setting program significantly increased daily steps and performance of timed up-and-go tests [14, 19]. These studies indicated that simply using the wearable devices may not change users’ behavior, accompanying intervention programs may be more important. Our previous study also disclosed that using pedometers among 440 community-dwelling older adults with some reinforcement from peers substantially motivated the willingness to adhere to the exercise programs and significantly improved walking speed for 0.06 m/s in 6 months [20].

The results of this current study further affirmed benefits of using wearable devices and extended the above-mentioned findings from the primary care setting or multimorbid participants to the healthy community-living adults. Our study findings suggested that active wearable devices were associated with 0.16 m/s faster walking speed than non-active users. A large pooled cohort study of 34,485 older adults reported that the incremental 0.1 m/s walking speed reduced 12% risk of 10-year mortality [5]. Among usual and active users, every incremental 1,000 average daily steps would increase 0.03 m/s of walking speed in 12 months. Moreover, booster walking steps of holiday warriors could improve walking speed for 0.5 m/s. The potential ceiling effects among active users may explain the differences of walking speed improvement between holiday warriors. In addition, serum levels of IGF-1 were positively associated with active wearable device users after adjustment for physical function (coefficient 22.9, p<0.001 for active users vs. non-active users). Our previous study showed that IGF-1 levels were significantly associated with muscle mass and its performance [21], which may suggest potential etiology for the health benefits of active wearable device use. In the study, gait speed was an independent measure from wearable device, whereas possible of using 5G to estimate gait speed in the future may provide a promising way to depict changes of walking.

Recent evidences linked slowness with impaired cognitive performance by shared neural substrate of hippocampus [22], and disrupted hippocampus-amygdala-cerebellum connections [23]. Although slow walking speed predicted the progression of cognitive declines [24], active wearable device utilization did not substantially improve cognitive performance. However, combined wearable device use and multidomain health promotion activities attenuated physio-cognitive declines of older adults [25], which may reduce the risk of disability, dementia and mortality in the long-term. The wearable device is more than an activity monitoring tool, but also a motivating factor to carry on healthy lifestyles [26]. The behavior of using wearable devices may not trigger the vigorous exercise to hinder cognitive decline, so the combination of multidomain health promotion activities is needed. Substantial dropping usage of wearable device suggested potential difficulties in sustaining benefits from the devices, in which reminders or motivation programs may enhance their adherence [20].

This study showed that participants with better cognitive function, independent of education years, were more likely to be active wearable device users. A recent study of 214 older adults aged ≥65 years in the United States showed that higher education and having fewer chronic conditions were positively associated with wearable device use [27]. Based on the technology acceptance model, perceived ease of use, perceived usefulness, users’ attitudes and behavioral intentions were all important factors for use of wearable devices [28]. Better cognitive performance facilitated older adults in comprehending the above-mentioned factors and tend to become active users. The average daily step counts of 6,284 among participants in this study were higher than previous studies, e.g. 5,138 in multimorbid obese participants, and 5,804 participants with chronic obstructive pulmonary disease [18, 29], but the conventional goal for daily 10,000 steps has been argued as unrealistic target for older people [30]. The current study showed an average daily step count of 7,000 may be a pragmatic goal for older adults, and was also the optimal target of 6,500-8,500 steps to prevent cardiovascular events [31].

Despite all efforts went in this study, there are still some limitations. First, the study did not collect information of personal interests for physical activities and leisure activities, which may confound the results collected from the wearable device. However, major determinants of health literacy such as age, education, and physical function were considered and full adjusted in our model as the previous study [32]. Second, increasing daily step counts were associated with faster walking, but the causal relationship remained unclear and the reverse causality may be a possible interpretation. Third, sex-specific analysis and other subgroup analysis were not conducted due to the limited sample size.

In conclusion, the study concluded the wearable device utilization patterns were associated with subsequent health benefits and increased the daily walking step counts and the walking speed. The benefits of active wearable device use deserve further attentions for the healthcare professionals and policymakers as the tool to promote healthy longevity.

## MATERIALS AND METHODS

### Participants and study design

This was a prospective cohort study conducted in Taipei City, Taiwan from November 2017 to April 2019. Participants were invited for participation if they met inclusion criteria as follows: 1) community-living adults aged between 50 and 85 years, 2) not having terminal illness or malignancy needing active treatment, and 3) able to communicate and sign the informed consent. All participants completed face-to-face interview and physical examinations for their demographic characteristics, past and personal history, and functional assessments by well-trained research staff. All participants were provided with the wearable device (Lifebeat, NeuroSky, Inc. CA, USA) to record physical activities by built-in pedometers and to estimate physical activities. The data were linked to the research team through the blue tooth transmission via their mobile phone application for further analysis.

The observational design and reporting format of this study followed STROBE guidelines [33]. The study design and procedures conformed to the principles of the Declaration of Helsinki. All participants were fully informed and provided written informed consent. The Institutional Review Board of National Yang-Ming University approved the study protocol (YM104121F-4).

### Parameters collected by the wearable device

All participants were categorized into non-active, usual and active users based on the utilization of their wearable devices and the completeness of data transmission to the research team. Those who used the wearable devices and completed data transmission for > 30 days in the follow-up period (at least once in both weekdays and weekend) were referred as active users; those used wearable devices for more than 3 days but not meeting the above-mentioned criteria were denoted as usual users [34]. Participants who only used the device in the first few days after enrollment without further data input were defined as non-active users.

Average step counts were defined as the mean of accumulated daily steps (per 1,000 steps) by the wearable device. Daily average step counts on weekday and holidays were recorded separately. Average daily steps ≥ 10,000 steps were defined as high daily steps [30], and the frequency of high weekday daily steps and holiday daily steps divided by all observed weekdays and holiday counts were defined as high weekday and holiday ratio, respectively. Those who had average daily step counts lower than one standard deviation of the mean, i.e., 3100 steps per day, were defined as low average daily step counts. Low weekday and holiday ratio of step counts were calculated accordingly.

### Outcomes

The main outcome of this study was the differences of usual walking speed during the study period, which was measured by a 6-meter walking test with non-accelerated start and non-decelerated stop. Participants walked at usual pacing along a flat, straight indoor space with a one-meter approach allowed before reaching the start mark and continuing walking past the end of six-meter path for a further meter. Research nurses timed their walking at baseline and 12 months later for comparisons. Secondary outcomes included handgrip strength, performance of 5-time sit-to-stand test and other functional tests. The maximum of three trials measured by a dominant hand at a sitting position with a flexion elbow was recorded as the handgrip strength. For 5-time sit-to-stand test, participants were instructed to stand up as quickly as possible for 5 times from the chair, and the research staff would time the process [35]. All participants also underwent serial functional assessments, including assessments for depressive symptoms, cognitive performance, physical function and nutritional status. The Center for Epidemiologic Studies Depression Scale (CES-D) was used to assess depressive symptoms and components for physical frailty [36]. The Functional Autonomy Measurement System (SMAF) was used to assess activities and instrumental activities of daily living [37], and a negative score indicated the presence of physical disability. The Montreal Cognitive Assessment (MoCA) was employed to evaluate the cognitive performance of all participants [38], and the short-form mini-nutritional assessment (MNA-SF) was used to assess for the nutritional status [39].

### Other variables

Demographic data including age, sex, education years, body height, body weight, health behaviors were carefully recorded by the research staff. The Charlson’s comorbidity index was used to estimate the diseases burden [40]. The average of three blood pressure readings under standard measurement procedures was taken for analysis. All participants received venous blood sampling after the 10-hour overnight fast. Serial biomarkers related to cardiometabolic health, hormones and biochemistry were tested for all participants, including fasting glucose, glycolated hemoglobin A1c (HbA1c), total cholesterol, triglyceride, and high density lipoprotein cholesterol (HDL-C), low density lipoprotein cholesterol (LDL-C), insulin level, HOMA-insulin resistance, and uric acid; inflammatory biomarkers (white blood cell, neutrophil to lymphocyte ratio, and platelet to lymphocyte ratio, homocysteine, and high sensitivity C reactive protein (hs-CRP)); age-related hormone (adrenocorticotropic hormone (ACTH), cortisol, growth hormone, insulin-like growth factor 1 (IGF-1), dehydroepiandrosterone-sulfate (DHEA-S), testosterone, sex hormone binding protein), and general biochemistry.

### Statistical analysis

Numerical variables were expressed as mean plus/minus standard deviation, and categorical variables were as number (percentage). One-way ANOVA was used to compare numerical differences across different status of wearable devices users, and chi square test was to compare categorical variables. Crude and multivariable linear regressions were used to explore associations between the use of wearable devices and changes of 12-month walking speed, handgrip strength, five times sit-to-stand test, SMAF, MNA-SF, CESD, MoCA and blood pressure measurements. Multivariable logistic regression was used to evaluate independent associated factors for the status of wearable device use. On those usual and active users, parameters of walking steps and patterns of physical activities were compared by student t test and multiple linear regression was used to evaluate their associations and changes of walking speed over 12 months. Youden’s index summarized the ROC analysis statistics was used to determine optimal average daily steps to prevent slowing walking speed [41].

For the analysis of using patterns of wearable devices, we used student t test to compare differences in individual parameters of wearable devices on usual and active users. Multivariable linear regression was used to explore associations between parameters of wearable devices and incremental walking speed among usual and active users. Stepwise multivariable multinomial logistic regression explored independent associated factors of usual and active users.

All analyses were performed using the SAS statistical package, version 9.4 (SAS Institute, Inc., Cary, NC, USA). A p-value from two-sided tests < 0.05, or 95% confidence intervals not spanning the null hypothesis values were considered statistically significant.

## Supplementary Material

Supplementary Table 1
