# Supplementary material for: Active wearable device utilization improved physical performance and IGF-1 among community-dwelling middle-aged and older adults: a 12-month prospective cohort study
Source: Aging (Albany NY). 2021 Aug 3;13(15):19710–21. doi: 10.18632/aging.203383 (PMC8386548; doi:10.18632/aging.203383)
Supplement: Supplementary Table 1 [file aging-13-203383-s001.pdf]

SUPPLEMENTARY TABLE

Supplementary Table 1. Multinomial logistic regression explored characters for wearable device users.

	Usual user(vs. non-active user)			Active user (vs. non-active user)		
	**Odds ratio**	**95% confidence interval**	**p**	**Odds ratio**	**95% confidence interval**	**p**
Stepwise multinomial logistic regression						
Education (years)	0.97	(0.88,1.07)	0.540	0.89	(0.80,0.98)	**0.022***
Systolic blood pressure(mmHg)	1.02	(1.00,1.04)	**0.025***	1.00	(0.98,1.02)	0.697
MoCA	1.27	(1.08,1.49)	**0.004****	1.28	(1.08,1.51)	**0.005****
ACTH (pmol/L)	0.95	(0.91,0.99)	**0.008****	0.97	(0.93,1.00)	0.077
Multinomial logistic regression adjusted for age, sex, education and Charlson comorbidity index						
MoCA	1.18	(1.03,1.36)	**0.020***	1.18	(1.02,1.36)	**0.028***
Systolic blood pressure(mmHg)	1.02	(1.00,1.03)	0.053	1.00	(0.98,1.01)	0.652
ACTH (pmol/L)	0.96	(0.93,0.99)	0.014	0.97	(0.94,1.01)	0.111

*denotes p<0.05; **denotes p<0.01, bold type denotes statistical significance. MoCA denotes the Montreal Cognitive Assessment ACTH denotes Adrenocorticotropic hormone.
